# A millisecond passive micromixer with low flow rate, low sample consumption and easy fabrication

**DOI:** 10.1038/s41598-021-99471-x

**Published:** 2021-10-11

**Authors:** Yuanyuan Liao, Yves Mechulam, Benedikt Lassalle-Kaiser

**Affiliations:** 1 Synchrotron SOLEIL, l’Orme des Merisiers, 91192 Gif-sur-Yvette, France; 2 Laboratoire de Biologie Structurale de la Cellule, BIOC, Ecole Polytechnique, CNRS, Institut Polytechnique de Paris, 91128 Palaiseau Cedex, France; 3 Present Address: IamFluidics BV, High Tech Factory, De Veldmaat 17, 7522 NM Enschede, The Netherlands

**Keywords:** Fluid dynamics, Lab-on-a-chip

## Abstract

Fast mixing of small volumes of solutions in microfluidic devices is essential for an accurate control and observation of the dynamics of a reaction in biological or chemical studies. It is often, however, a challenging task, as the Reynolds number (*Re*) in microscopic devices is typically < 100. In this report, we detail a novel mixer based on the “staggered herring bone” (SHB) pattern and “split-recombination” strategies with an optimized geometry, the periodic rotation of the flow structure can be controlled and recombined in a way that the vortices and phase shifts of the flow induce intertwined lamellar structures, thus increasing the contact surface and enhancing mixing. The optimization improves the mixing while using a low flow rate, hence a small volume for mixing and moderate pressure drops. The performances of the patterns were first simulated using COMSOL Multiphysics under different operating conditions. The simulation indicates that at very low flow rate (1–12 µL·min^−1^) and *Re* (3.3–40), as well as a very small working volume (~ 3 nL), a very good mixing (~ 98%) can be achieved in the ms time range (4.5–78 ms). The most promising design was then visualized experimentally, showing results that are consistent with the outcomes of the simulations. Importantly, the devices were fabricated using a classical soft-lithography method, as opposed to additive manufacturing often used to generate complex mixing structures. This new device minimizes the sample consumption and could therefore be applied for studies using precious samples.

## Introduction

Microfabricated devices based on the “lab-on-a-chip” concept are increasingly found in the fields of biology^1,2^, analytical chemistry^3^, and medicine^4,5^, especially during the last two decades. This concept is based on the use of microfluidic platforms to study fundamental processes, and therefore requires highly controlled experimental conditions. The miniaturization of an entire analytical system was initially dedicated to significantly reduce the working volume to a range of microlitres or even picolitres^6–8^, and/or increase the efficiency of heat or mass transfer. Following, with the progress in microfluidic/electrical device fabrication techniques and materials, lab-on-chip devices were developed to perform many other functions, such as trapping^9,10^, sorting^11,12^, filtration^13,14^ and spraying^15–17^. Currently, multi-functional chips are increasingly becoming a focal point in the field.

Among all the functionalities that microfluidic devices can offer, obtaining a controllable, homogeneous mixing of the fluids is of particular interest, especially for fundamental chemical/biological kinetic studies, or when fast diagnosis is required. However, liquid flow within a micron-scale channel is typically laminar and the mixing process depends mainly on molecular diffusion. This process is very slow, especially in the case of macromolecules such as proteins, typically with low diffusion coefficient < 10^–11^ m^2^ s^−1^, and therefore limits kinetic studies to slow processes.

Another challenge for the mixer is to limit the sample consumption, as most applications use very high flow rates (300–3000 µL·min^−1^) to achieve a fast mixing. These high flow rates are not only excessive for many valuable samples that are produced at the µL scale, but also requires special equipment and complexity in the chip fabrication to handle the high pressures required. Most of the micromixer that allows operating with high flow rates are fabricated in a solid material and need to withstand pressures higher than 5 bars^6,18^.

In order to reduce the mixing time to, or even below, the ms range, numerous mixing devices have been designed and developed. Those mixers are typically classified into two categories: active, and passive micromixers^19–22^. Active micromixers are often externally driven, for example by using acoustics^23–25^, electric fields^26,27^, or magneto-hydrodynamics^28^. In general, active micromixers have better mixing efficiencies, but are more complex to fabricate. Moreover, external power sources might damage or modify the biological samples dissolved in the liquids^21^. Passive micromixers are, on the other hand, usually easier to fabricate and control, because they only require the input pressure to drive the flow.

The mixing efficiency of a passive micromixer relies mainly on molecular diffusion^7,19^, which is related to the diffusion coefficient, the interfacial surface area and the concentration gradient. With the diffusion coefficient often constrained by the sample and the experimental conditions, the design strategy consists in maximizing the latter two factors. Optimizing the configuration of the fluid channel can be used to induce fluid stretching, folding or breakup. A variety of novel design concepts of micromixers based on this principle have been investigated elsewhere^7,21,22^. Among them, chaotic advection-based micromixers emerged as a potential solution that can not only enhance the mixing, but also reduce the required channel length. The concept is to introduce microfeatures such as barriers and restrictions in the channel, which can generate transverse flows^29–35^, hence promoting “chaotic advection”. The method is particularly well suited for fluidic systems with low Reynolds number.

Staggered herringbone micromixers (SHMs) take advantage of chaotic advection to achieve mixing. The concept is based on inserting oriented grooves on one or both walls of the microchannel to induce whirls and transverse flows that enhance the diffusion among fluids^29–31,36–38^. The mixing efficiency of SHB structure is typically investigated using a number of SHB cycles required to complete mixing^30,31^. The most efficient design of one cycle is composed of two sets of grooves (equal number of grooves per each set) asymmetric with respect to the center axis, and the long arm of the herringbone structure is 45 degrees with respect to the axial direction of the channel^31,39^. The geometry will create elliptic vortices flowing over a helical path, the number and the arrangement of the grooves controlling the rotation angular displacement of the fluid. This displacement of the flow is often defined by the measure of the amplitude of the rotation in half-cycle (one set of grooves), ΔΦ^31^.

The method works well for fluids with very low Reynolds numbers (~ 1), requiring only a modestly higher pressure than a standard straight channel^21,22^. It significantly enhances the efficiency and the homogeneity of the mixing while providing a very uniform residence time distribution^39^. The SHM concept was first proposed by Stroock et al*.*^31,40^, where the angle between cycles was fixed at 90°. This design choice was adopted in numerous studies^41^, and has been widely studied and improved^29,42–46^. Improvements were made by modifying the shape and the pattern of the groove^43^, the depth and the inclination (angle between the groove and the channel) of the groove^44^, the number of subsections to complete a mixing cycle^45,46^, or the arrangement of the grooves features, such as the effects of using positive or negative grooves^29^. However, all those studies focused only on SHMs based on T or Y shaped mixing channels, which do not optimally use the vortices created in the fluids.

In this paper, we detail a SHM combined with a double T-junction to realize the pre-mixing of two aqueous solutions, followed by several in-channel ring-type elements. The two fluids were split into four streams and premixed 2 by 2 separately on both sides, and then further mixed in the repeated ring-shaped units. As indicated earlier, with the arrangement of SHB, the rotation angular displacement of the fluids on both sides can be altered in a way that the circulating flows will encounter in the mid-zone with the maximum interface and recombine in the main channel. The recombined fluids will go through the vertical mixing cycle and then go to the repeated ring-shaped microunits. The ring-shaped unit was designed not only to reduce the working volume, but also to subsequently introduce the mixed solution to an observation channel or nozzle of similar size. The repeated curved microchannel also enhances the mixing with the introduction of the transverse Dean flows across the channel due to the centrifugal effect^47,48^.

We report in this paper the design, fabrication and experimental testing of this family of devices, as well as the theoretical interpretation of their mixing mechanism. Several arrangements of the direction of the grooves with the double T junction were designed using SOLIDWORKS. The mixing behaviors and efficiencies of SHMs were simulated using COMSOL Multiphysics. The simulation results were used as a guide to design the chips, by quantifying the mixing efficiency at cross-section and time-dependent concentration distribution. The most efficient design with the partition stagger herring bone (SHB) cycles was selected and its performances were confirmed by experimental observation.

## Materials and methods

### Device design and fabrication

**Figure 1:**
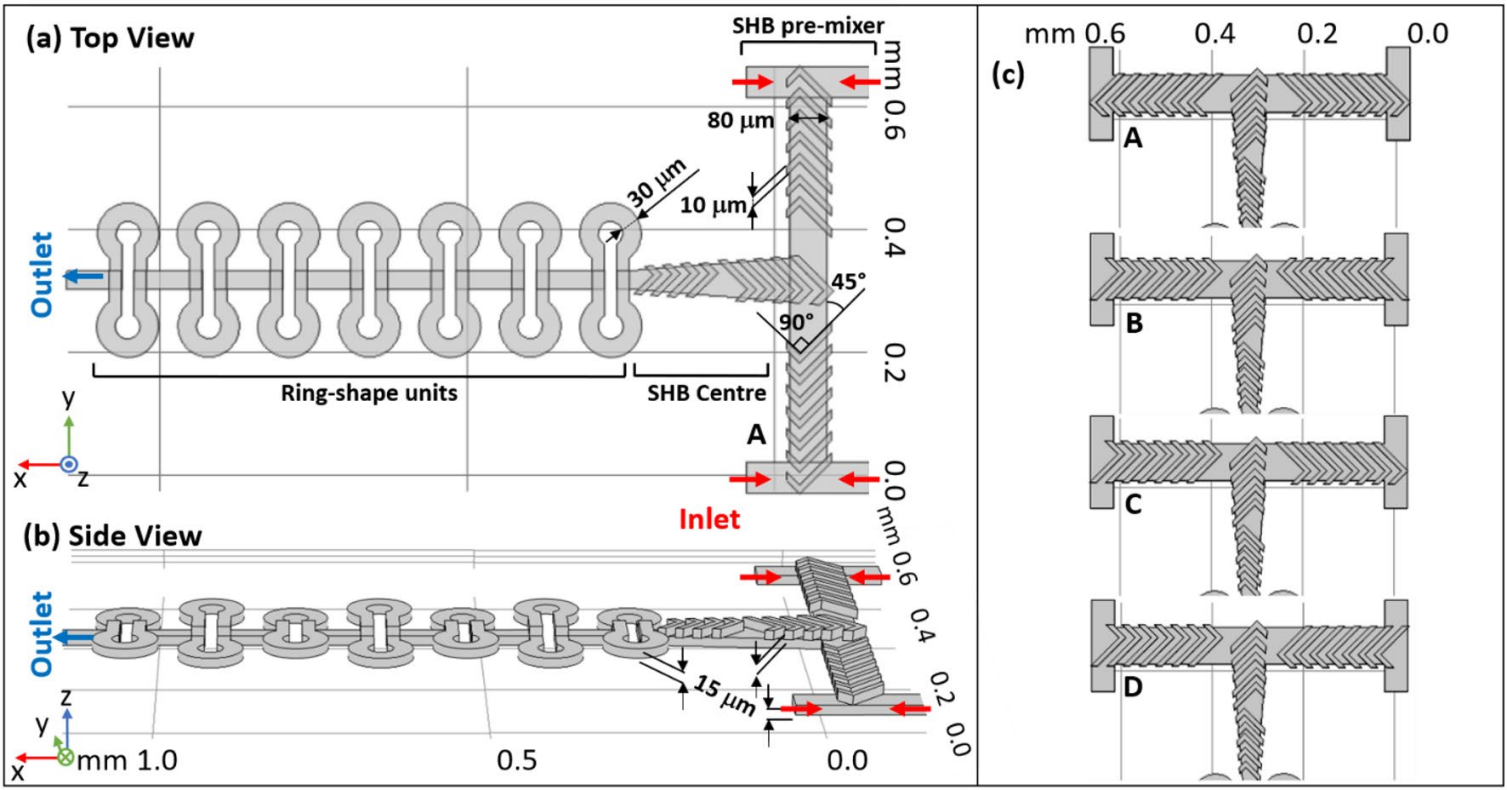
The structure details of the complete mixing compartment studied in this paper in top **(a)** and side views **(b)**. The mixer is composed of three zones: SHB pre-mixer, SHB center channel and ring-shape units. The mixer region is built out of two separate layers: the top one contains the SHB ridges and the bottom one the double-T pre-mixer channel. Several ring-shaped units are also combined with the main channel on the bottom layer during fabrication. **(c)** The four different arrangements of the SHB including the one shown in **(a,b)**, for a total of four SHB designs for the double T structure, marked A, B, C and D. The 3D sketches shown on this figure were made with the SOLIDWORKS 2020 software (http://www.solidworks.com).

The design was first conceived with SOLIDWORKS, with four inlets, two on the right side for one fluid and the other two on the left for another fluid (Fig. 1a). Two miscible fluids are first premixed in the first T entrance before reaching the second T-junction element. The inlets are of 50 µm width, and the width of the main channel of the second-T junction is 80 µm. The basic concept of the SHB geometries is taken from Stroock et al*.*^31,40^, with the grooves geometry parameters complying with the size of the our main channel. In this experiment, the number of grooves on each side of the T-junction are related to a half cycle of the classic SHM design^31,40^, where one cycle of the SHM is composed of 7 + 7 short and long asymmetric grooves. Both negative grooves and positive grooves of 10 µm width and 15 µm depth, 45° to the floor channel were studied. The entire volume of the mixing zone presented in Fig. 1 (including the 7 ring-shaped units) is ~ 3.5 nL, which is several orders of magnitude smaller than the typical SHM design with a volume in the µL range^30,31,37^. The size of the mixer is comparable to the mixer destined to a similar use in the study by Lu et al*.*^6^*,* in which a very high flow rate (300 µL·min^−1^) was applied to achieve fast mixing (ms range) in the same volume. A recent study^49^ employing a more complex 3D mixing design used two-photon stereolithography to produce a chip that allows homogeneous mixing in a similar time range, while maintaining the mixing volume at ~ 3nL. In this case, the fabrication strategy appears as the limiting factor due to accessibility and cost of such a 3D printer.

Figure 1c shows four pattern designs with the SHB spanning over the double-T junction in different orientations related to the flow direction: The standard SHB shape A is indicated as “Forward”^30,31^ and the inverted shape B is indicated as “Reverse”. Also, in symmetric or asymmetric arrangements of the SHB on both T-side: A, B are with SHB “axial symmetry”, C is “centrosymmetric” and D is “asymmetry”. The rationale behind these different arrangements, is that the vortex of the flow induced by the SHB ridges can be altered by modifying the direction or the symmetry of the ridges on each side, resulting in different counter faces in the center, where the flow recombined and enters the repeating units. To the authors’ knowledge, the principle of using the rotation flow induced by SHB to create sequential-lamination has never been used elsewhere, and it can enhance the mixing efficiency strongly in a simple and elegant way.

A circle of 5 + 5 grooves covers the channel that is 90º to the first T-junction. It naturally scales down the mixing channel before entering the ring-shaped region. When the channel is narrowed down to 30 µm, the grooves are difficult to fabricate, thus a curved channel design was applied here to take advantage of the channel complexity. The design is based on the Dean-vortex micromixer^48^, which creates vortices from the flow field phenomena in curved channels. With this design, the curvature of the channel amplifies the lateral instability, deducing a secondary cross-channel flow so as to enhance the mixing. Repeating the curved feature intensifies the Dean flow effect, thus further improving the mixing^47^. Our design uses rings with a 30 µm width and a radius of curvature of 30 µm as well.

**Figure 2:**
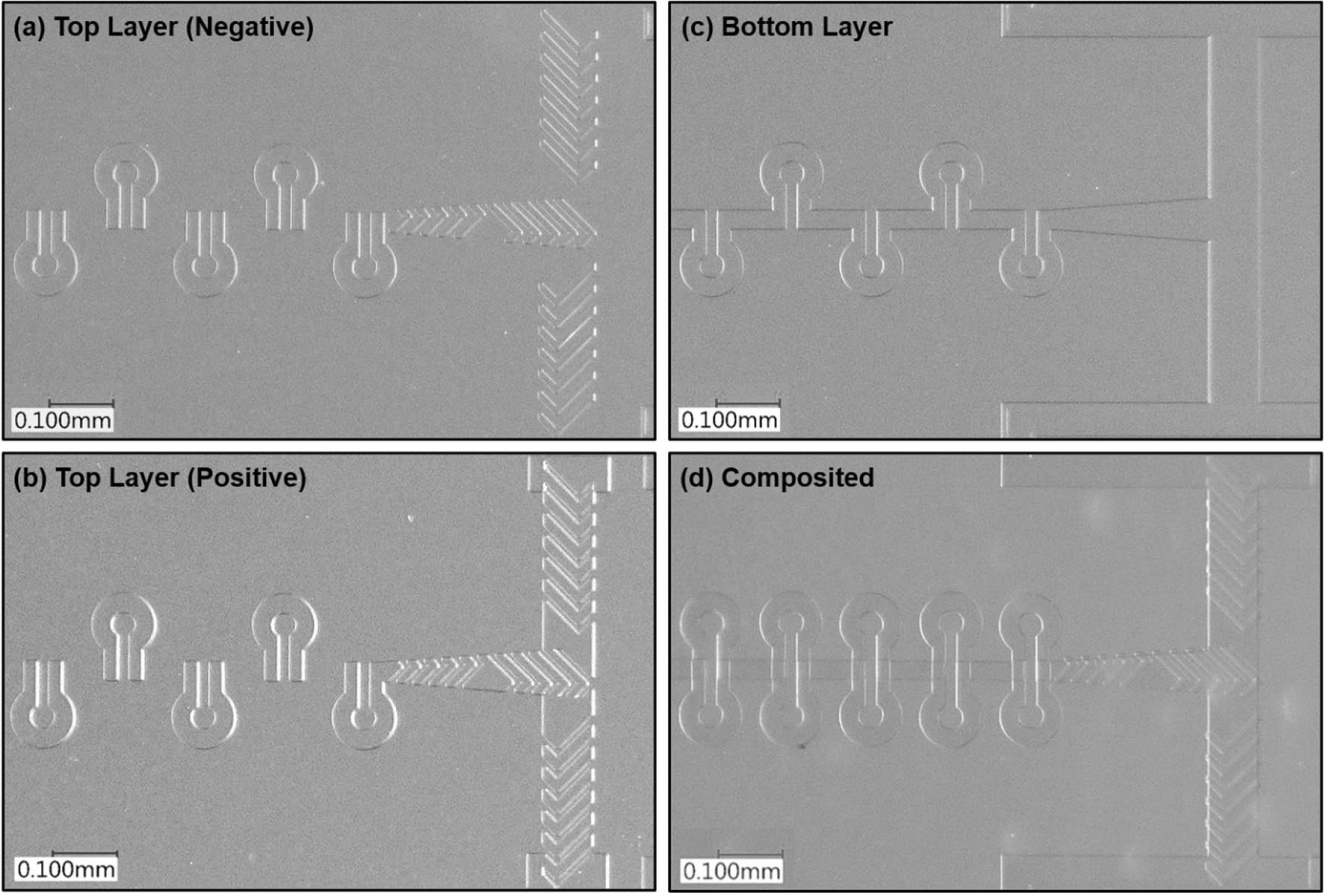
White light microscopy images of SU-8 mold of pattern C top layer with negative grooves **(a)**, PDMS replicas of pattern C positive grooves of top **(b)** and bottom **(c)** layers. **(d)** is an example of the composite micromixer fabricated by plasma bonding PDMS replicas **(a)** and **(c)**. All images were taken with a × 20 microscope objective.

All microfluidic chips used in this study are fabricated using polydimethylsiloxane (PDMS, Sylgard 184 silicone elastomer, DOW CHEMICALS). Our design of the mixer required two SU-8 masters, one for the top layers with SHB units, and one for the bottom layer for the main channel. The two masters are realized by fabricating the related microstructure systems on a polished silicon wafer using commercial negative photoresisting SU-8 (SU-8 3010, MICROCHEM CORP.). The mixer zone contains only one layer on each side. All channels and patterns are defined by maskless UV laser photolithography system (KLOE, Dilase-250)^49^. The fabricating steps are explained in SI. Figure 2 shows the top layer (a, b) and the bottom layer (c) PDMS replicas obtained from the master molds respectively. The two layers will be well aligned, and plasma bonded together (d).

### Experimental setup and mixing measurement

The mixer-device was mounted onto a microscope stage and samples were pumped through the device using a two-channel syringe pump (NE-4002X, NEW ERA Pump Systems, Farmingdale, NY USA). The liquids were flowed using the syringe pump and two glass syringes of 500 µL size (HAMILTON Gastight syringe, 1700 series) with PTFE Luer lock and Teflon plunger. Solutions of selected indicators were introduced with optimized flow rates, and fluorescence images of the mixing zone were taken to characterize the fluid behavior in the microfluidic device.

Rhodamine B conjugated to 70 kDa dextran was selected to be the indicator in this study, since the corresponding diffusivity (~ 2.68 × 10^–11^ m^2^/s)^50^ is very similar to the one of ribosomes (2.1 × 10^–11^ m^2^/s)^51^.

The mixing behaviors of all the patterns selected were first observed by mixing Dextran-Rhodamine B conjugate with a Fluorescein aqueous solution. The fluorescence intensity of Dextran-Rhodamine B conjugate and Fluorescein are sensitive to the pH of the solvent, which needs to be considered^52^. Two indicator aqueous solutions, one of 10^–5^ M Dextran 70 kDa-Rhodamine B conjugate and another of 5 × 10^−6^ M Fluorescein, were injected into the four inlets of the mixer separately. All solutions were prepared using filtered distilled water (0.45 mm pore size) to prevent dust clogging inside the channel. The fluorescence images of the mixing process obtained are composed by two-color channels and compared to the simulation result using COMSOL. On the other hand, when mixing Rhodamine-dextran with fluorescein in the device, the intensity of the signal collected by each channel can be complicated by the crossover of their fluorescence emission band. Thus, the mixing efficiency (the time required for a mixing of *ca.* 95%) was evaluated by mixing Dextran 70 kDa-Rhodamine B with distilled water only. The brightness profiles were extracted before and after each repeated ring-shaped unit along the channel following the flow and compared to the brightness profile at the entrance of each channel before mixing started.

All the fluorescence micrographs were acquired with an inverted fluorescence microscope (Olympus IX83-CBH) with a ZDC module, a Märzhäuser stage (Scan IM 120 × 80), an HXP120 light source, and an OrcaFlash4 v3 camera (Hamamatsu). The snapshot with different focusing plane and the switch of the lamps were auto controlled by the Micromanager software (version 1.4.21). The fluorescent dyes were excited using the standard FITC (excitation: 475 nm, emission: 530 nm) and TRITC filter set (excitation: 542 nm, emission: 620 nm) with exposure time 100 ms and 400 ms separately, the 10 × magnification objective lens was applied (UPLFLN10 × PH2, NA 0.3). Images were taken and stored as image stacks and analyzed using ImageJ image analysis software (National Institute of Health, USA). The background is removed using an average of the brightness of 200 pixel using ROI selection.

## Numerical simulation

The mixing performance of all the designs were evaluated and compared using COMSOL Multiphysics 5.5, following stationary steady-state mixing and time-dependent studies with “Particle Tracing for Fluid Flow (FPT)” and “Transport of Diluted Species (TDS)” modules respectively. Physics-controlled mesh with coarser element size is applied to generate mesh by the software to meet the need of the calculation and comply with the computation capacities of a desktop computer.

### Velocity field analysis

Initially, a stationary study was applied to all the designs to solve the mixing in steady state, with a portioning into ~ 7 × 10^4^ elements and an average element size of ~ 0.6 µm depending on the micromixer geometries. A finer mesh yields a higher resolution of the simulation; however, it also requires a higher demand for computation, so here the mesh degree was selected to fit within the available computational capacities. The study included two steps: first, the velocity and the pressure fields in the mixer were solved by using the Navier–Stokes’s Eqs. (1,2) for an incompressible Newtonian fluid built in the module of “Laminar Flow (SPF)”.

1 $$\left(\rho \frac{\partial {\varvec{u}}}{\partial t}-\nabla \cdot \left[\left(-p\right)I+\mu \left(\nabla {\varvec{u}}+{\left(\nabla {\varvec{u}}\right)}^{T}\right)\right]+\rho {\varvec{u}}\cdot \nabla {\varvec{u}}\right)=F$$

2 $$-\nabla \cdot {\varvec{u}}=0$$

where **u** is the velocity vector of the flow (m·s^-1^), $\rho$ is the fluid density (kg·m^-3^) and *p* is the pressure (Pa), I is the unit diagonal matrix, µ is the fluid’s dynamic viscosity (Pa·s), and F is the bulk force affecting the fluids (N)^53^. The equations are solved for a steady flow with each of the inlet flows set as laminar flow. The boundary condition at the inlets was set initially to a uniform velocity profile, with the mean velocity *v* of the inlets’ flow calculated from the preset flow rates employed throughout the study. Other boundary conditions, such as the wall condition is “zero slip” at the solid surfaces, and the pressure of the outlet channel is set to 0.

The mean flow velocity *v* that applied initially at the inlets of the system were also key parameters to characterize the flow regions, and it corresponds to the Reynolds number, *Re*.

3 $$Re=\frac{\rho vL}{\mu }$$

Here, $\rho$ is the liquid density, $\mu$ is dynamic viscosity, and *L* the hydraulic length of the channel. Obviously, increasing *v* increases *Re* and reduces the influence of the inertia effect. Flow rates ranging from 1 to 12 µL·min^−1^ were applied to each of the four inlets to the mixer system, yielding a mean velocity at the entrance boundary from ~ 0.02 to ~ 0.26 m·s^−1^. The corresponding *Re* in the main channel of the T mixer region ranges from ~ 0.78 to 9.4. When both side flows are recombined in the repeated ring-shaped units, the *Re* number increases to a value from ~ 3.3 to 40.

To characterize the mixing efficiency of a passive type mixer, other than the Re number, another dimensionless value is worth to discuss, which is the *Péclet* number, *Pe*. The *Re* number is often used to categorize the operation range for different mixers of which the designs are based on chaotic advection, while the *Pe* number is used to characterize the required mixing length, which can be obtained by $${\raise0.7ex\hbox{${\nu L}$} \!\mathord{\left/ {\vphantom {{\nu L} D}}\right.\kern-\nulldelimiterspace} \!\lower0.7ex\hbox{$D$}}$$.

The flow rates applied here can be easily achieved with syringe pumps specialized for microfluidics (NE-4002X, New Era Pump Systems, Farmingdale, NY USA). If the flow rates are increased to 60 µL·min^−1^, the *Re* number in the ring-shaped region will be ~ 200, which means that the transverse motion of the fluid becomes more significant and stirs the flow more rapidly, so as to enhance the mixing by increasing the interfacial area between the two fluids. But with such high flow rate, the pressure inside the channel will also be very high, increasing the complexity of the experiment. In particular, this increases the requirements of the equipment in terms of driven force, rigidity of the chips and liquid inlet connections. Automatically, the change would increase the volume of liquid required for mixing.

### Convection–diffusion analysis

Subsequently the obtained velocity profile will be stored in the solution and then used to calculate the concentration distribution by convection–diffusion Eq. (4), using transport of diluted species (TDS) physics.

4 $$D{\nabla }^{2}c-{\varvec{u}}\cdot \nabla c=0$$

where *c* is the concentration, *D* is the diffusion coefficient and ***u*** is the velocity vector field obtained from the previous study. The experiments and simulations were carried out at room temperature, with diffusion coefficients for water (*D*_*water*_) and the solute (*D*) set to 2.29 × 10^–9^ m^2^/s^54^ and 2.1 × 10^–11^ m^2^/s, respectively. The diffusion coefficient *D* is the room temperature diffusion coefficient of 30S ribosomes^51^, which simulates the potential use of this mixer for macromolecular kinetic studies. The concentration was set to be 1 × 10^–6^ M.

Another parameter that is used to quantify the convection–diffusion terms is the *Péclet* number, *Pe,* which can be defined as $uL/D$. Mixing is diffusion-dominated for small *Pe* and convection-dominated for large *Pe.* In this study, the *Pe* is much larger than 1, so the mixing will be convection-dominated.

To evaluate the efficiency and the homogeneity of the fluids mixing, the mixing index was defined here and calculated at several locations following the SHB structures and also before/after every ring-shaped element. The mixing index was defined using the standard deviation of the concentration fraction extracted from the cross-section image taken at all the specified locations. The variance of the concentration can be obtained by Eq. (5)^6,55^:

5 $${\sigma }^{2}=\frac{1}{N}\sum_{i=1}^{N}{({c}_{i}-\overline{c })}^{2}$$

where N is the total number of points taken from the line plot for analysis (depending on the mesh elements), ${c}_{i}$ is the local concentration at point *i*, and $\overline{c }$ is the average of the concentration over the area of interest.

Then the mixing index γ can be calculated with Eq. (6)^6,55^:

6 $$\gamma =\left(1-\sqrt{\frac{{\sigma }^{2}}{{\sigma }_{max}^{2}}}\right)\times 100\%$$

where ${\sigma }_{max}$ is the maximum standard deviation with fluids unmixed at the entrance of the inlets, where the mixing index γ should be 0%. The mixing index should be between 0 and 100% through the mixing, a value of 100% indicating that a fully mixed, homogeneous solution is obtained.

Then, a time-dependent study was applied for a chip chosen from the previous steady-state study based on its mixing properties. By carefully choosing a time range and time steps, one can observe the development of the steady-state concentration profile and determine the approximate mixing time.

### Particle tracing analysis

Another time-dependent study with “Particle Tracing for Fluid Flow (FPT)” module was also implemented based on the velocity profile obtained previously. The study aims to evaluate the mixing performance by calculate the transportation of the suspension particles during the in-line mixing from a Lagrangian point of view. With the study of particle tracing, the motions for particles based on the flow field can be calculated by computing the drag forces (Eqs. 7, 8), where the ${{\varvec{F}}}_{{\varvec{D}}}$ is the drag force, ${{\varvec{m}}}_{{\varvec{p}}}$ is the mass of the particle, ${{\varvec{\rho}}}_{{\varvec{p}}}$ and ${{\varvec{d}}}_{{\varvec{p}}}$ are the density and the diameter of the particle separately.

7 $$\frac{d}{dt}\left({m}_{p}v\right)={m}_{p}{F}_{D}\left({\varvec{u}}-v\right)$$

8 $${F}_{D}=\frac{18\mu }{{\rho }_{p}{d}_{p}^{2}}$$

We applied 8000 particles released from the inlets of the mixer at the beginning, with all the particles uniformly distributed at the four inlets boundary surface. The time intervals of the calculation is given by $$5 \times 10^{{ - 6}} \;{\raise0.7ex\hbox{${\text{m}}$} \!\mathord{\left/ {\vphantom {{\text{m}} {\text{v}}}}\right.\kern-\nulldelimiterspace} \!\lower0.7ex\hbox{${\text{v}}$}}$$, which is carefully selected to comply with the velocity of the particles, so as to avoid losing particles^46^ in particle trajectories simulation. The average velocity $$v$$ applied at each inlet is calculated from a flow rate of 6 µ min^−1^, and with such flow rate, the particles should be passing though the outlets in ~ 9 ms. Thus, the simulation time is set to 9 ms to allow all the particles travelling through the geometry that was carried out for this simulation. In this study, only three repeated-ring units was involved in the calculation. Although a larger number of particles with a shorter time interval can offer a more accurate visualization of the fluids, however it also makes the computation more expensive and harder to complete. $${\rho }_{p}$$ was set to be 1200 kg·m^−3^ with $${d}_{p}$$ = 7.4 nm, the parameters are proximately the density and diameter of Dextran 70K^56^. To visualize the distribution of the particles during the mixing, the Poincare maps which is the cross-section of the particle trajectories, is plotted along the channel. The color of the particles indicates their original location, to be more specific, the four different inlets where they entered. The results can be helpful to visualize the motion of the flow process in the channel with the changing geometry, the lamination due to the rotation effect^57^.

## Results and discussion

### Study 1

**Figure 3:**
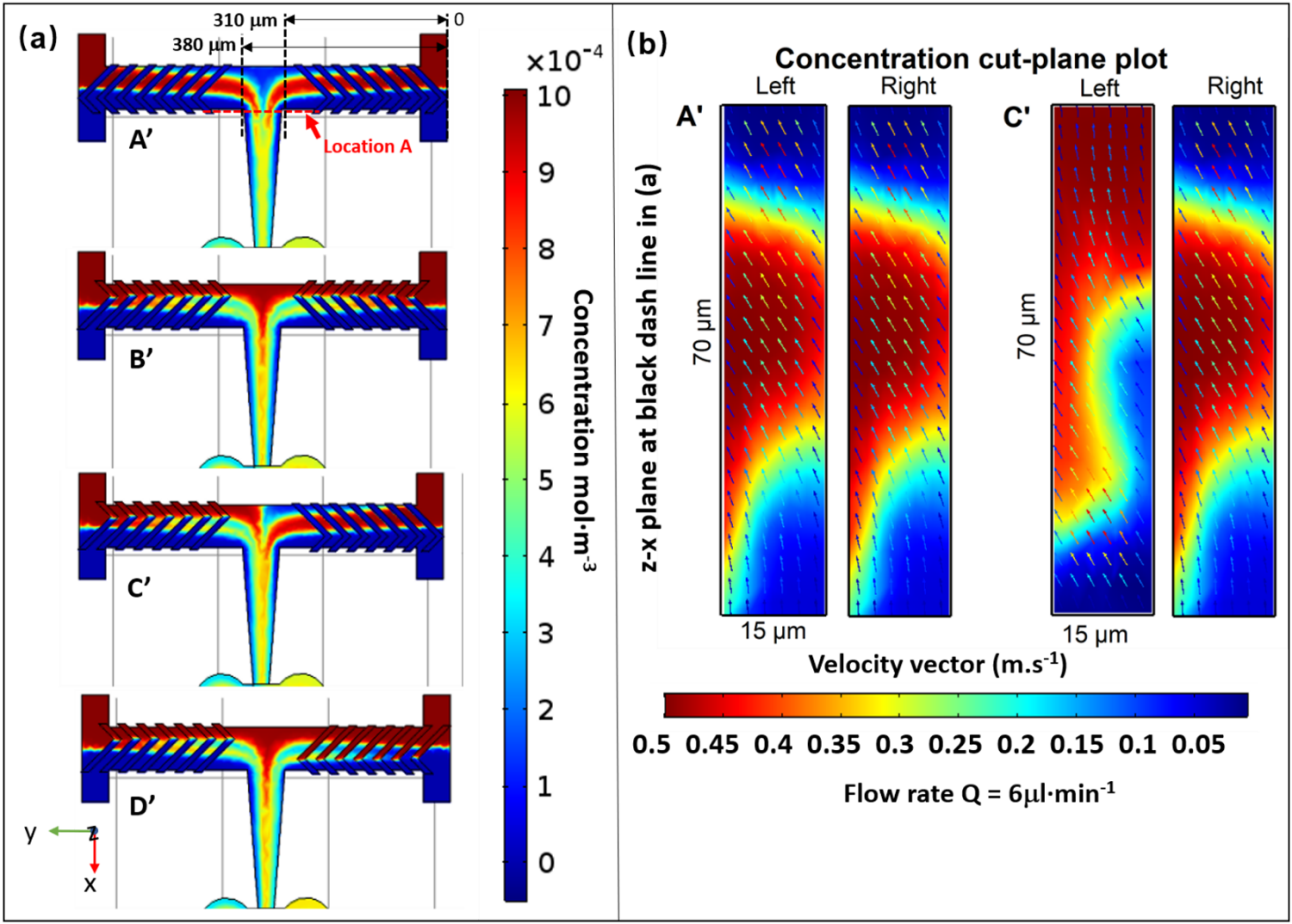
Simulation results along the mixing channel of four SHB designs A’, B’ C’ D’. **(a)** shows the concentration profiles in the *x–y* plane at *z* = 7.5 µm (middle of the channel), the two black dashed lines indicate the locations at the end of premixing after passing through half cycle of the grooves on each side (distance of 310 µm and 380 µm from the entrance at the right), while the red dashed line indicates the location where the combined solutions change the flow direction to *x*. **(b)** is the slice concentration profile projected onto *x–z* plane located at the black dashed lines at 310 and 380 µm from the right channel labelled as “left” and “right”, respectively. The concentration profile shares the same color bar with **(a)**. Velocity vectors were plotted as colored arrows with the color bar representing the velocity (m·s^−1^). The simulations were performed with the COMSOL Multiphyics software (http://www.comsol.com).

This section analyzes the concentration field of the mixers experimentally and numerically. Figure 3 provides concentration profile of four designs A’, B’, C’, D’, which contained only half a mixing cycle (7 grooves of same direction) on each side of the double T pre-mixer channel corresponding to the designs A, B, C, D presented in Fig. 1. The grooves in the center channel have been removed in order to provide a better vision of the concentration profile at the interfacial surface, so as to see how the direction of the grooves on both sides is involved in enhancing the mixing efficiency.

Simulations were performed with flow rates of 6 µL·min^−1^ at each inlet, giving a *Re* value of *ca.* 4.7 in the horizontal pre-mixer channel, with the initial concentration of the solute set to 1 × 10^–3^ mol·m^3^. Figure 3a shows the concentration profile of the slice projected onto the x–y plane in the middle of the main channel. The colors closer to red or blue indicate less mixing, while green corresponds to a better mixing. The angle between the grooves and the channels walls creates a transverse flow on both side of the double T pre-mixer, which rotates the stream of the two fluids and thereby induces mixing through the channel. Before the two incoming channels meet, a symmetric or asymmetric rotation pattern of the fluids in counter flow direction can be clearly seen. For A’ and B’, as the grooves on both sides are in mirrored configuration, it yields a mirrored concentration distribution and velocity profile, as can be seen in Fig. 3a. On the other hand, the C’ pattern, with the asymmetric arrangement of the grooves on each side, causes the two fluids to meet with vortices with opposing torque before they meet (Fig. 3b).

At the black dashed line location where the fluids recombine, and as one can see for A’ and C’, the multi-lamination is quite obvious. The B’ and D’ patterns, however, do not seem to induce a particular effect on the mixing. To have a better view of this effect, the concentration profiles were extracted in the *x–z* plane at two symmetrical locations right after the flow passing through the half-cycle of the grooves, at the black dashed line with a distance of 310 (right) and 380 µm (left). The obtained profiles are presented in Fig. 3b, where the distortion feature of the concentration is more obvious. After the two flows coming from opposite sides recombine in the center, the flow direction changes 90 degrees. The folding of the phase shifts streams in a lamellar manner due to the rotation effect, and creates more interfacial areas, which is the main strategy to enhance the mixing in this study.

There are four interfaces created for the A’ and C’ patterns, while only three are created in the B’ and D’ designs. Only two would be created in a typical T-shaped design after a half cycle rotation.

**Figure 4:**
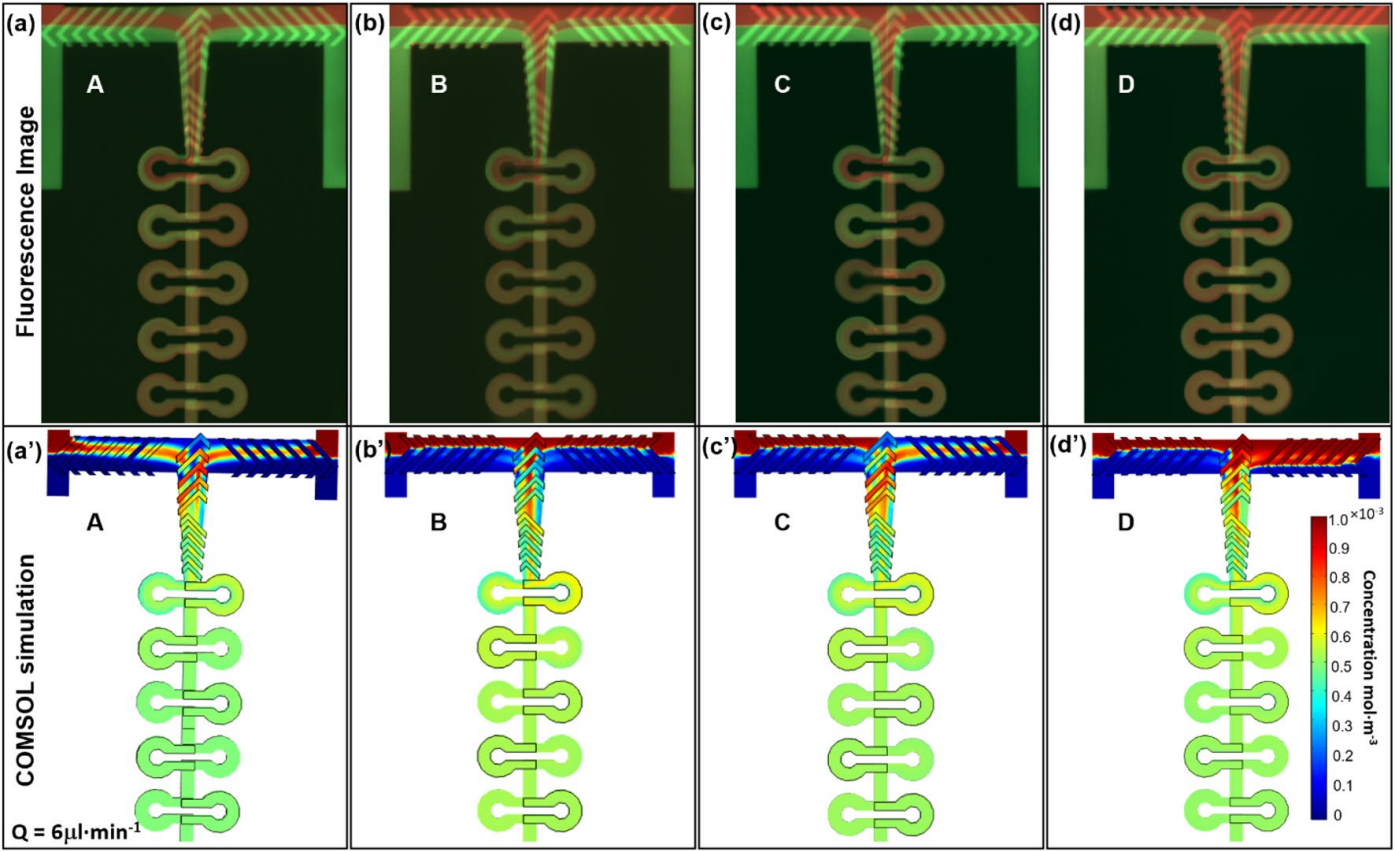
Fluorescence microscopy images (top: **a–d**) of the mixing zones in devices with 4 selected SHB designs A, B, C and D, with their corresponding simulation results (bottom: **a’–d’**). Rhodamine B conjugate 70 kDa dextran (red) and Fluorescein (green) are used for fluorescence visualization, with flow rates Q for each inlet of 6 µL·min^−1^ and *Re ca.* 20 in the repeated ring-shaped units.

To better demonstrate the rotation pattern of two aqueous fluids, we observed the mixing using two indicators, Dextran-Rhodamine B conjugate and Fluorescein. The results are presented in Fig. 4.The fluorescence images and the corresponding simulations of all four A, B, C and D SHB designs (see Fig. 1) are shown in Fig. 4. Both experimental and computational data show the mixing status in the double-T junction and in the first 5 repeated ring-shaped units. The geometry of the PDMS microchannel can be clearly seen and the region observed contains around 2.93 nL. The flow rate is 6 µL·min^−1^ for each inlet, to reduce the consumption of the sample for a given time. The different evolution of the mixing patterns obtained by the experiments are consistent with the simulation results. Initially the two fluids are combined at the entrance from both sides and rotate in the same or the opposite direction controlled by the direction of the grooves, then at the first vertical turn, the “lamination” of the flow at the second T junction appears and is clearly due to the groove geometries and flow directions.

Typically, the average diffusion time *t* of a macromolecule in a micron-sized channel is proportional to the square of the diffusion distance: $$t\propto {\raise0.7ex\hbox{${x^{2} }$} \!\mathord{\left/ {\vphantom {{x^{2} } {2D}}}\right.\kern-\nulldelimiterspace} \!\lower0.7ex\hbox{${2D}$}}$$, where *x* is the width of the channel and *D* is the diffusion coefficient. The lamination of the flow reduces the distance by the number of laminae it creates, therefore, the mixing time is reduced by a factor of the laminae number squared. For designs A and C, the water flow can be considered as being subdivided into three streams while is subdivided into two in the other designs (B and D). The mixing will be much faster (four times) and more homogeneous with three flows (four laminae) than with two flows (two laminae). The rotation behavior also creates a larger interface compared to a normal split-combined stream, due to the stretching and distortion of the flow and the larger surface of the laminae.

For a split-and-recombine (SAR) mixer based on a “multi-lamination” mechanism, the main problem is often related to implementing multiple inlet channels, rotation effects or a splitting-recombination strategy, which typically adds to the complexity and expense of the design^34,58^. A strategic improvement would be Baker’s transformation mechanism^59^, by introducing an intricate 3D structure to split the flow, then rotate and change the direction of the flows before recombining/folding them^60,61^. The purpose is always the same: generating an intertwined lamellar structure. Inspired by a herringbone structure rotating effect, our device mimics Baker’s transformation mechanism without introducing more inlets or increasing the complexity of the geometry, thus providing a more elegant and technically simpler way to achieve the same purpose. The simulation of the flow behavior (Fig. 4a’–d’) shows a very good agreement with experimental results. The symmetry and asymmetry concentration profiles at the double T location are consistent in experimental and simulation results.

After the pre-mixing double T-junction, the liquid will have a 90º sharp turn and sway along the repeated curved channel, separating and recombining several times until a complete mixing is achieved. With this design, the mixing will be improved by transverse Dean flows developed in curved channels due to the action of the centrifugal force. The principle is the following: in this circularly shaped channel, the flow will be bent in 3D, and the centrifugal force will pull the inner stream to the outer wall radially, forcing the fluid originally located close to the outer wall to move laterally inwards through the top and bottom of the channel based on the conserve mass principle^62,63^. The generated transverse flow causes an exponential growth of the interfacial area, which significantly improves the mixing. The same phenomenon also applies when the flow turns 90º at each T junctions entering or exiting the ring-units.

Such effect can be observed experimentally in Fig. 4a,c. One can see that when entering the first ring unit, the red stream appears close to the inner wall on the left side, but close to the outer wall on the right side. After the curved channel, in response to the centrifugal force, the red stream swept towards the opposite side when recombined with the stream in the reverse manner, coming from the left-side ring. Starting from the second ring unit, multiple interfaces can be found on both left and right curved channels.

Comparing the experimental results with the simulation results in the ring-units region, a similar concentration profile can be observed, but the orientation of the flow is hard to trace. This is why the calculation of the fluidic particle trajectories can be helpful, which will be presented later. Also, it appears clearly that the mixing efficiencies after the first pair of ring-units are different among those four patterns. The A design presents the best mixing, with a green color indicating that the solute concentration is close to 0.5 × 10^–3^ M, which is half of the initial concentration. The B, C and D designs present a color closer to yellow, which means that the mixing is not as good as for the A design. In general, the A pattern gives a better mixing efficiency compared to the other designs.

### Study 2

**Figure 5:**
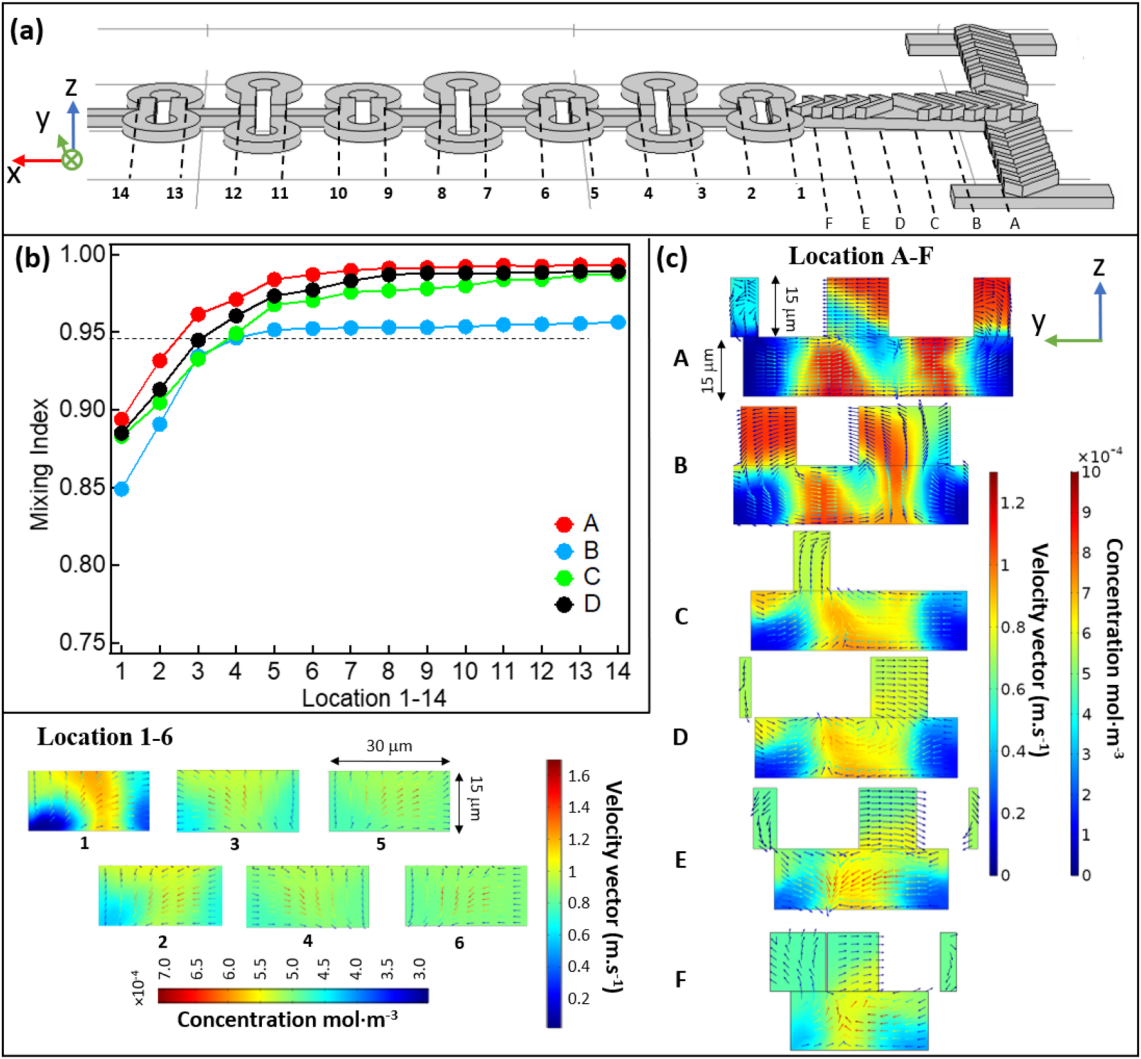
Represents the simulation results along the mixing channel of all the four designs at Q = 6 µL·min^−1^, *Re* ~ 4.7 in pre-mixer and ~ 20 in ring-shaped units. **(a)** side view of the mixer pattern A with position marks in the main mixing channel and in the ring-shaped mixing zone. **(b)** Mixing index data obtained by simulation of the evaluated sample for all four cases in Fig. 4, same conditions were applied. The concentration data chosen here for the mixing indices calculation were extracted from the concentration profile projected onto the z–y plane at the locations labelled 1–6 on the mixing channel of panel **(a)**. **(c)** shows the slice concentration profile projected onto the z–y plane at the locations labelled A–F on the mixing channel of panel **(a)**, with the distributions of the velocity vectors overlayed on the same profile. The color bars for the concentration profile are presented besides, with the concentration ranging from 0 to 10 × 10^–4^ mol·m^−3^. The simulations were performed with the COMSOL Multiphysics software (https://www.comsol.com).

In this section, the mixing efficiency of each design was further evaluated by simulations based on the concentration profiles at different locations, and comparison to experimental results. The same flow rates were applied for the sake of consistency with the previous study. Representative results are shown in Fig. 5.

The mixing index were calculated using Eq. (6), with the concentration profiles extracted at each location 1 to 14 in the *yz*-plane, before and after all repeated ring units in Fig. 5a. The obtained results were plotted versus the location index presented in Fig. 5b. With the flow rate Q = 6 µL·min^−1^ for each inlet, in all cases the mixing index achieved 95% or even higher by location 6, which means that the required working volume for a complete mixing is around 2.5 nL. Under these conditions, the time required for a complete mixing is *ca.* 7 ms. Apparently, with the A, C and D designs, mixing is clearly more efficient than for the B design. The relatively shorter mixing time observed for A and C is due to the multi-lamination effect illustrated previously. In the case of D, it could be due to the counter-rotating vortices created on both sides, increasing the interfacial surface area.

Figure 5c shows the simulated concentration distribution for pattern A at the cross-sections from location A to F, and location 1 to 6, as labelled in Fig. 5a. After the pre-mixing in the first T-junction, the flow from both sides of the channel encounter and turn 90º to enter the central channel. The frames extracted at location A and B clearly show the rotation and distortion of the flow on the y–z plane, which is perpendicular to the main flow direction (x-direction). The velocity vectors plot was overlayed with the concentration profile to illustrate the mixing effects, where the color bar for velocity vector indicates the speed of the fluid at a given point. With the velocity vectors plot, one can find three vortices at the cross-section of location A: two rotate in clockwise direction in the middle and one on the right; one rotates in counter-clockwise direction on the left. Compared to the classic Y-type SHB mixer^30,31^, liquid flowing from both sides and going through half a circle of SHB before entering the central channel will obviously create more interfacial areas. Combined with the velocity vectors in Fig. 3b, one can see clearly how the two species counter-rotate, wrapping each other following the flow direction A to F. Furthermore, from the concentration profiles A to F (Fig. 5c) we can see that the fluid lamellas are not equal in thickness, the lamellas next to the channel wall are clearly thicker than the one in the middle. Also, the mixing is faster in the center compared to the edge, which derives from the increased size of the lamellas next to the channel walls. This is due to the lower flow velocity in the vicinity of the walls, which is indicated by the color bar of the velocity vector. Actually, the non-uniform mixing at the edges is often observed in classical SHB microfluidic channels (Fig. S2)^30^.

In this case, changing the flow direction by introducing ring-shaped units can push the center flow to the channel wall and subsequently improve the mixing homogeneity across the channel. Before entering the ring-shaped units, the concentration profile at the cross-section of the flow is presented in F (Fig. 5c). Then the flow splits in the middle and enters the right circle and left circle separately.

In our design, when the applied flow rate is in the range of 1 to 12 µL·min^−1^, the corresponding *Re* is less than 10 in the pre-mixing double-T region, which is a low *Re* value (< 10). When the flows from both sides recombine and enter the ring-shaped region, the center channel narrows down and the *Re* is increased to 3.3–39.8, reaching an intermediate *Re* range (10 to 100)^47^. It has been proved that SHB mixers operate independently of the *Re* number, which makes this type of design especially favorable in low flow rates condition. On the other hands, the mixer designs based on Dean vortex requires much higher *Re* numbers, approximately above 150. In fact, in our *Re* range (3.3–39.8), the Dean vortices will appear in a symmetrical manner with both streams staying in their original half mixing channel, the inertial forces not being strong enough to move the inner stream towards the outer stream of the ring-shaped channel. The existence of vortices due to secondary flow caused by centrifugal force can only been observed at the sharp 90º turn of each entrance, where the required *Re* number for Dean vortices is lower than in circular shape. If the flow rate increases four times (48 µL·min^−1^), thus increasing the *Re* number to 160, the mixing efficiency will be largely enhanced because the symmetry in the Dean vortices will be destroyed and real chaotic advection will appear. This is why in many other applications for fast mixing, a high flow rate is essential for operation, with values of at least 60 µL·min^−1^^6,64^. With such a high flow rate, the pressure drop inside of the device introduces complexity in the fabrication, and the sample consumption rate will increase dramatically, making *in-situ* experiments impossible over long times.

With the velocity of the flow applied in this experiment, a very large *Pe* is unavoidable (> 10^5^). This means that the convective transport is dominant. In this case, the characteristic mixing length for a classical parallel laminar mixing will be very long, equals to *PeL *(> 1 m), as the downward flow path of the solute dominates the transversal diffusion path. When Q = 6 µL·min^−1^, which can be considered as a modest flow rate, the *Pe* in this micromixer is in the order of 10^5^. It gives ~ 2 × 10^5^ in the first T junction and ~ 4 × 10^5^ in the second T junction, due to the increasing speed of the flow caused by the recombination of the streams. When the channel narrows down before entering the repeated ring-shaped channel, the *Pe* is even higher, i.e. ca. 10^6^. In a mixer without optimization to enhance mixing, the characteristic mixing length can reach 10 m under the same conditions. However, with chaotic mixing, the required mixing channel length can be reduced largely due to the exponential stretching of the interfacial area as mentioned earlier, and a well-known expectation of mixing length in this case would be ~ *w ln(Pe)*^31^, where *w* is the width of the channel and typically in hundreds of µm range. The estimation is confirmed by several experiments, with the mixer adopting the herringbone structure or other designs. The experimental mixing length is typically several tens of times the estimated distance. Stoock et al*.* show that with a classic herringbone Y mixer under similar conditions (Pe ~ 10^5^, v ~ 0.1 m/s), the mixing distance obtained by experiments gives ~ 1.5 cm, while the estimated mixing distance would be ~ 2.2 mm (width of the channel, *w* ~ 200 µm), which is roughly 6.8 times less^31^. He suggests a SHM could achieve full mixing of all flows with *Pe* < 10^6^ with a channel of 3 cm, which is around 11 times of the estimated mixing distance. Other studies with SHM type mixers that fall within this *Pe* range typically require 3 full circles to achieve full mixing, as different numbers of grooves and distances of inter-circle were applied. The characteristic mixing distance is in a range of 5.9 to 7.6 mm, roughly 30 times their geometry^29,30^, giving a factor of 3.0 to 3.8 of the estimated value. In our case, the estimated mixing distance calculated from the *Pe* number will be ~ 1.1 mm, while the experiment result gives 1.5 mm, which gives a factor of 1.4. However, none of the studies mentioned above is working with volumes in the nL range, but rather in the µL range (0.5 µL). We have studied the classical herringbone designs and translated the conditions in which they were previously tested to our smaller design. The results are consistent with what these designs should yield on a smaller scale, and our device demonstrably improves the mixing efficiency with respect to these classical designs, expressed in a shorter mixing distance. This is elaborated on in the SI (Fig. S3).

Apparently, in our study, this mixing distance is largely reduced again due to the rotating lamination effect, thus achieving mixing with only a few nL.

**Figure 6:**
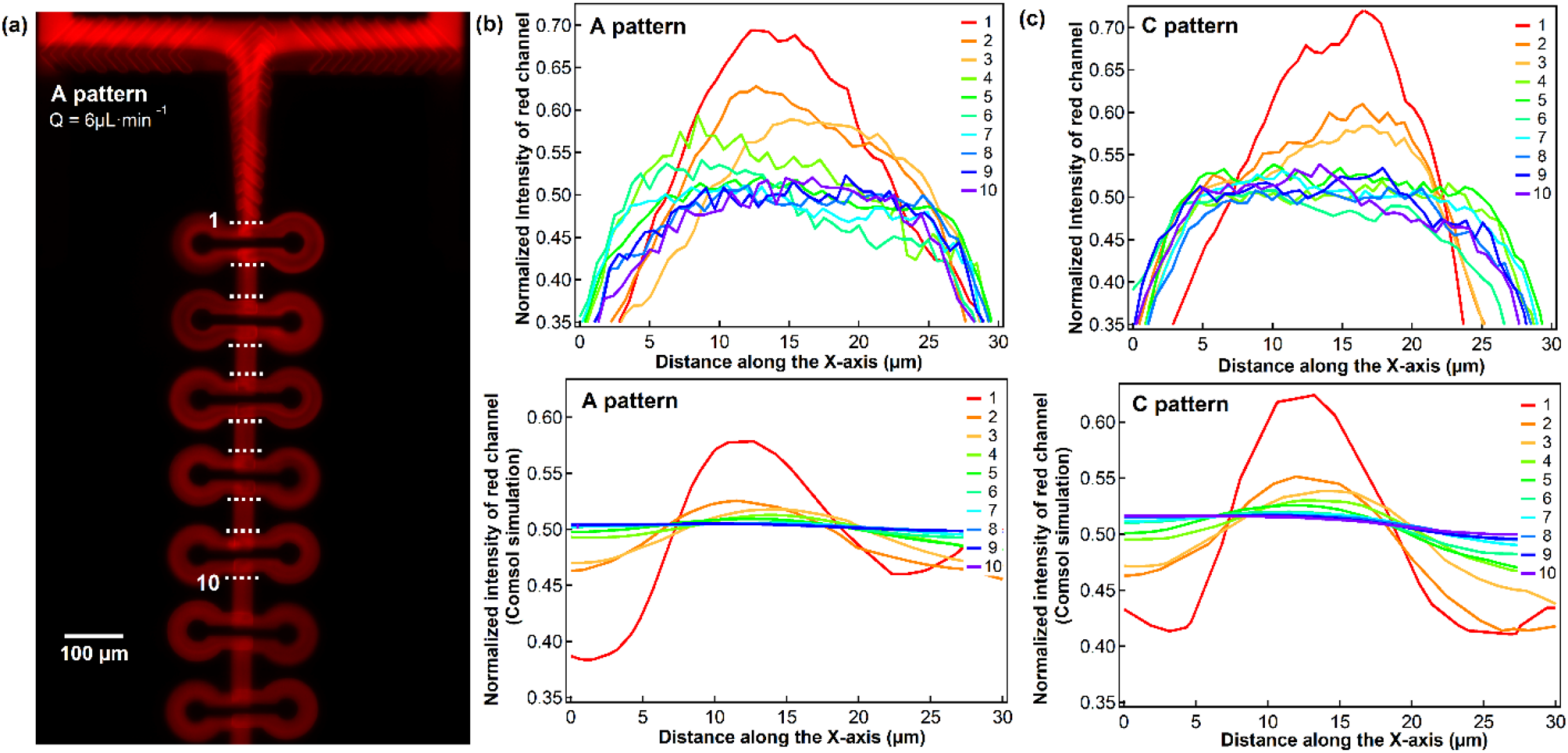
Mixing of an aqueous solution of Rhodamine B-dextran 70 kDa with distilled water. **(a)** fluorescence image of design A, performed with the same parameters as in Fig. 4, **(b)** the experimental Dextran-Rhodamine B conjugate concentration profiles across the channel at the indicated location in **(a)** (dash line) compared to the COMSOL simulation results at the same location. The same experimental concentration profiles and simulation results for pattern C were plotted accordingly in **(c)**.

The mixing efficiency was further evaluated by mixing a Rhodamine B conjugated to 70 kDa dextran aqueous solution with distilled water. In this study we use a top-down fluorescence microscope to assess the degree of mixing. The captured fluorescence microscopy image for pattern A with a flow rate of 6 µL·min^−1^ is shown in Fig. 6a. The experiment was repeated with all four patterns described above, and the corresponding intensity profiles across the channel at location 1 to 10 (dashed line in Fig. 6a) for pattern A and C are shown in the top panels of Fig. 6b,c respectively. These results are compared with the theoretical intensity profiles at the same location obtained with COMSOL, which are shown in the bottom panels of Fig. 6b,c. Experimentally, the degree of mixing is characterized by the concentration, which should be half of the initial one for a complete mixing. This corresponds to normalized intensity set to 1 at the inlet for the Rhodamine B solution on Fig. 6a. The fluorescence signal at each location was then normalized to 1 and plotted versus the position across the channel.

With the given flow rates, experiments show that 4 to 5 ring-shaped units are sufficient to achieve a complete mixing (normalized intensity of 0.5–0.55). As we are using thin PDMS layers on one side to compensate the working distance of the object lens, the parabolic shape of the intensity close to the channel walls is attributed to the deformation of the channel caused by the high pressure.

The experimental results match the simulation results well, with a minimum number of ring units of 2–3 to reach a normalized intensity below 0.5. The mixing efficiency seems to be slightly overestimated by COMSOL, however, with a computational normalized intensity of 0.52 after 2 rings in pattern A, while the experimental value is 0.64. This discrepancy is less pronounced for pattern C and the overall trend is nevertheless reproduced for both patterns. It is worth mentioning that the fluid thinner than the minimum resolution of COMSOL cannot be distinguished, which is related to the computational mesh size of *ca.* 0.6 µm, and an experimental resolution of 0.5 µm for the fluorescence camera (Laterally). It derives that, especially for high *Pe* number, an insufficient mesh resolution could lead to artificial or inaccurate results of COMSOL. A recommended way of calibrating the mesh to gain the right balance between performance and numerical stability can be found in Hadjigeorgiou et al.^65^.

### Study 3

In this section, we are characterizing the mixing properties with a time-dependent study using particle tracing analysis and convection–diffusion analysis.

**Figure 7:**
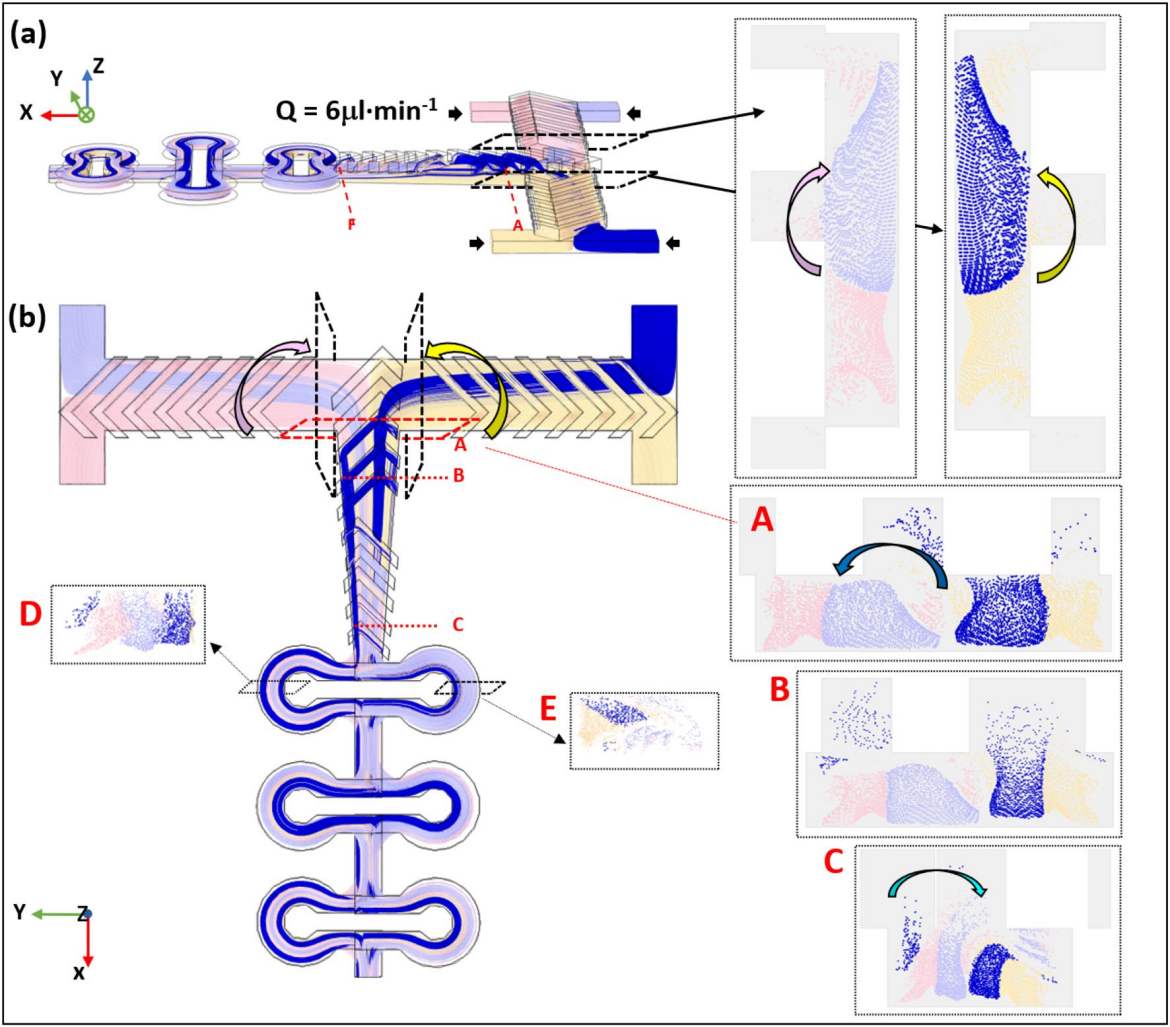
Particle trajectories along the mixing channel from side **(a)** and top **(b)** views. Poincaré maps of different cross-section at locations A–E along the channel are shown in the insets.

The simulation study with particle tracing module of the COMSOL Multiphysics software is presented here (Fig. 7), using the same flow rates of Q = 6 µL·min^−1^ applied in the former study. The simulation results present the transport properties of the mixer from a Lagrangian point of view. The four different colors of the particles indicate their original location, i.e. the four inlets where the particles originate from. The light blue and the blue colors can be considered as one fluid, while the yellow and the pink colors presents another fluid.

Figure 7 presents the Poincaré maps showing the distribution and the density of the particles for locations A to E through the cross-section plane along the mixing channel.

It appears clearly that the herringbone feature causes the fluids to continuously twist and rotate. In the top view, (Fig. 7b) the fluid entering from the bottom two inlets (yellow and pink colors) bend and move above the fluid that enters from the top two inlets (blue color), before they both split into two streams. Then, from location A to B, the fluid bend from right to left and left to right, with the dark blue stream rotating and splitting into two streams, and the light blue stream splitting into two streams at location C. The Poincaré maps explain how the lamination happens and matches the concentration profiles (Fig. 5) perfectly. At locations D and E, a uniform mixing of the two fluids can be observed, which proves that we have a highly efficient mixing. Sometimes the particles get stuck to the channel walls where the magnitude of flow velocity is low, and it could explain why the number of the particles is progressively reduced during the mixing as can be seen from the cross-sections at locations A to E.

Finally, a time-dependent study was done to calculate the approximate mixing time necessary for different flow rates with device A (see SI). The simulations were applied with flow rate values of Q equal to 1, 6 and 12 µL·min^−1^, the corresponding *Re* in the ring-shaped region being 3.3, 20 and 40 respectively (4 × Q). Only the snapshots for Q = 6 µL·min^−1^ are presented in Fig. S1, as in the current flow rates range (1–12 µL·min^−1^), *Re* is still in the intermediate range (10–100) and the simulation results are roughly the same. Thus, the time required for sufficient mixing for the three flow rates are inversely proportional to the chosen flow rates, and the required mixing distance is relatively invariant. The second snapshot (t = 8.5) was chosen when the flow had just passed location 6 (after the third ring), at which point we demonstrated above that mixing is achieved at 98%. The times required for such a mixing for Q = 1 µL·min^−1^ and 12 µL·min^−1^ are 52 ms and 4.5 ms, respectively. The last snapshot is set to the moment when the flow travelled through the whole channel.

## Conclusion

In a microfluidic device, mixing is often limited to molecular diffusion due to the laminar nature of the flow and becomes a challenge when mixing macromolecules or biological samples with large diffusion coefficients. Another challenge is to keep a balance between the consumption of sample (driven by the flow rate) and a fast, homogeneous mixing, while avoiding complex and costly device fabrication.

Although many micromixers have been developed for viscous samples with mixing in the ms range^6^; none of them exhibit the mixing efficiency at low flow rates demonstrated in our device. The reason for that is their geometries is not able to provide sufficient chaotic advection with a low *Re* but only functional at very high flow rates.^6,64^ The micromixers that have been reported so far fall in either one of the two following categories: those that either require much longer times (subsecond or seconds) and/or much larger working volume (several µL) to achieve mixing^29,30^, or use a very high flow rate (5 µL/s, Re ~ 200) to achieve mixing in the ms time range. On the contrary, our device contains < 3 nL volume, and it can achieve a complete mixing in the ms range while applying a low flow rate (4 × 6 µL·min^−1^ for 8.5 ms), which strongly reduces the sample consumption and opens up opportunities for studies involving precious samples such as membrane proteins or metalloenzymes. The mixing efficiencies of devices with positive SHB designs were also studied and the results are presented in the SI (Fig. S4). The conclusion is very similar as for the negative one, although the working volume is approximately twice of the negative design. The devices described in this study can be fabricated with a classic soft-lithography method using PDMS, which is cheap and convenient as compared to two-photon stereolithography or 3D printing techniques with micron-sized resolution. It therefore provides a fast and affordable option to achieve mixing of precious chemical or biological samples within milliseconds and with very low volume consumption^64^.

All data generated or analysed during this study are included in this published article (and its Supplementary Information files).

## Supplementary Information

Supplementary Figures.
