# Supplementary material for: A millisecond passive micromixer with low flow rate, low sample consumption and easy fabrication
Source: Sci Rep. 2021 Oct 11;11:20119. doi: 10.1038/s41598-021-99471-x (PMC8505571; doi:10.1038/s41598-021-99471-x)
Supplement: Supplementary file 1 — Supplementary Figures. [file 41598_2021_99471_MOESM1_ESM.docx]

A millisecond passive micromixer with low flow rate, low sample consumption and easy fabrication.

Electronic Supplementary Information (ESI)

Yuanyuan. Liao ^1,*^, Yves. Mechulam^2^, and Benedikt. Lassalle-Kaiser^1,*^

^1^ Synchrotron Soleil, l’Orme des Merisiers, Saint-Aubin 91192 Cedex, France

^2^ Laboratoire de Biologie Structurale de la Cellule, BIOC, Ecole polytechnique, CNRS, Institut Polytechnique de Paris, Palaiseau cedex, 91128, France.

[^*^benedikt.lassalle@synchrotron-soleil.fr](mailto:*benedikt.lassalle@synchrotron-soleil.fr)

**Table of contents**

1. **Mixing device fabrication procedure**
2. **Figure S1.** Simulation results of time-dependent studies of the mixing in the channel for design A.
3. **Figure S2.** Details of a positive SHB mixer studied in this paper.
4. **Figure S3.** Simulation results of a classic herring-bone structure with negative grooves mixer.
5. **Figure S4.** Representative fluorescence microscopy images corresponding to mixing in devices with 4 possitive SHB designs.
6. **Mixing device fabrication procedure**

**Dilase mask-less fabrication method**. The master for the reported microfluidic mixer chips is realized by fabricating the related microstructure systems on a polished silicon wafer using commercial negative photoresist SU-8 3010 (MICROCHEM) wherein the microfluidic channels are defined by UV-laser photolithography. The fabrication process is schematically demonstrated as follows:

1. The design of the microstructure is created through Solidworks and its pattern .Dxf file are processed by the software KLOE DESIGN, which associate the original design to a .lwo file containing the laser trajectories. Then, the obtained .lwo files are transmitted to the Dilase printer (Dilase 250, KLOE).
2. The silicon wafer is spin coated with a 15 mm thickness SU-8 photoresist. After soft baking at 65 °C for 1 min and 2 min at 95 °C, it is then transferred to the Dilase machine. A UV laser beam with a size of 4 µm is used for direct writing on the silicon wafer following the laser trajectories in the .lwo file, then the silicon wafer is transferred to a hot plate at 65 °C for 1 min and 2 min at 95 °C for post bake.
3. After the post bake, the wafer should be cooled down to room temperature first, then immersed in SU8 Developer (MICROCHEM) to remove the unexposed materials. Next, the silicon wafer master is rinsed subsequently with isopropanol, acetone and water.
4. With the obtained master wafer, the next step is to produce the replica out of it. The polydimethylsiloxane (PDMS) was prepared by mixing the standard elastomer base and curing agent in a ratio of 10:1. This well mixed PDMS gel was degassed in a vacuum chamber for 30 mins, then it was poured onto the master mold and baked for 2h at 80 °C. After cooling down to room temperature, the cured PDMS replica can be easily removed. The details of the mixer geometries are presented in Fig. S1 and Fig. 1. Two replicas, one containing the herring-bone feature, the other one the main channel, are delicately aligned under a microscope and plasma bonded in the center (Fig. 2).

**Figure S1.** Simulation results of time-dependent studies of the mixing in the channel for design A. Time evolution of mixing for Q = 6 μL·min^-1^, Re ~ 20 in the ring-shaped region. Snapshots were taken at t = 1 ms, 8.5 ms and 13 ms. The simulations were performed with the COMSOL Multiphyics software ([www.comsol.com](http://www.comsol.com)).


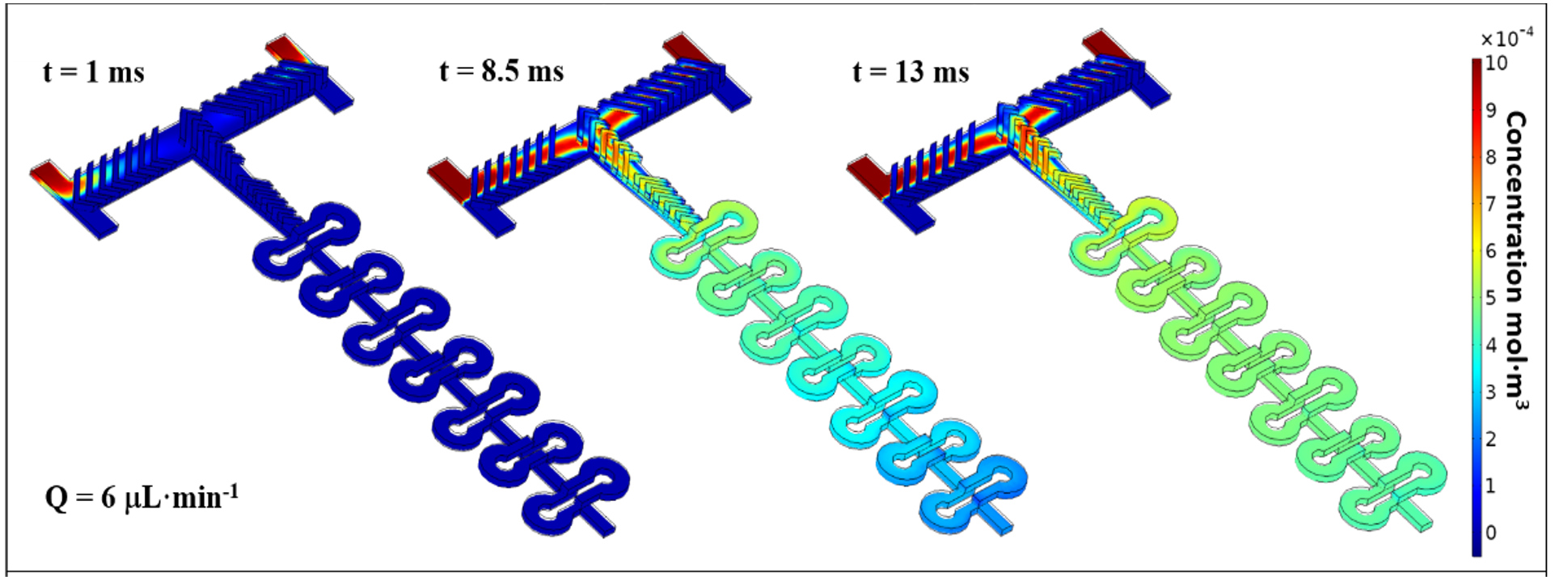


**
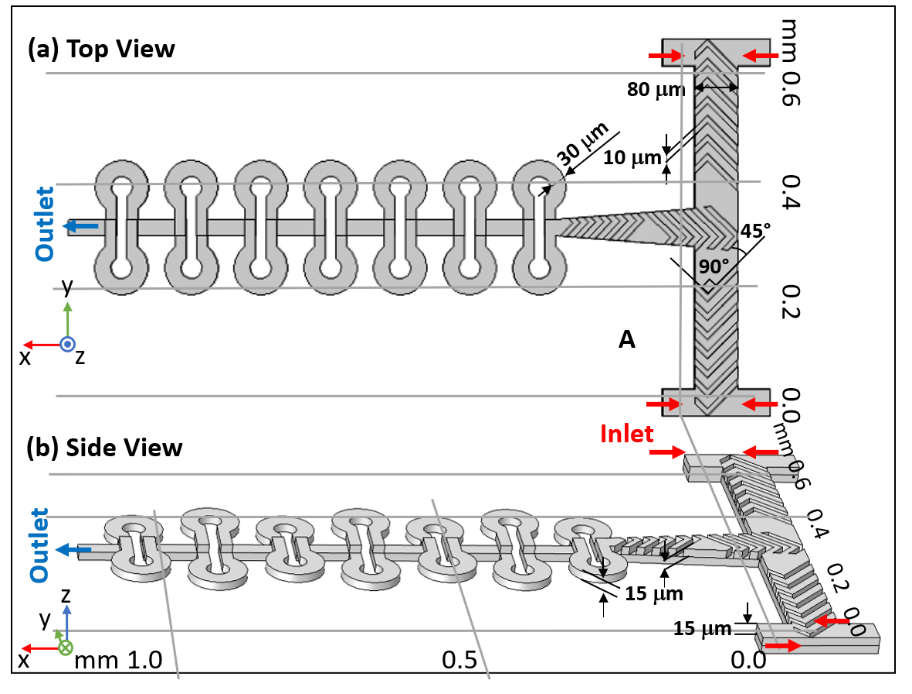
**

**Figure S2.** Details of a positive SHB mixer in top view **(a)** and side view **(b)** studied in this paper. The mixer region comprises two separated layers. The top layer contains positive SHB ridges over the double-T premixer channel (bottom layer), also with several ring-shaped units to be combined with the main channel at the bottom layer in fabrication.

Figure S2 shows the geometry details of a positive SHB mixer with a symmetry arrangement of the grooves on both side of the T junction. The positive SHB mixer has convex grooves spreading over the main channel, with its groove’s parameters, the thickness of the channel is the same as the conventional negative SHB mixer.


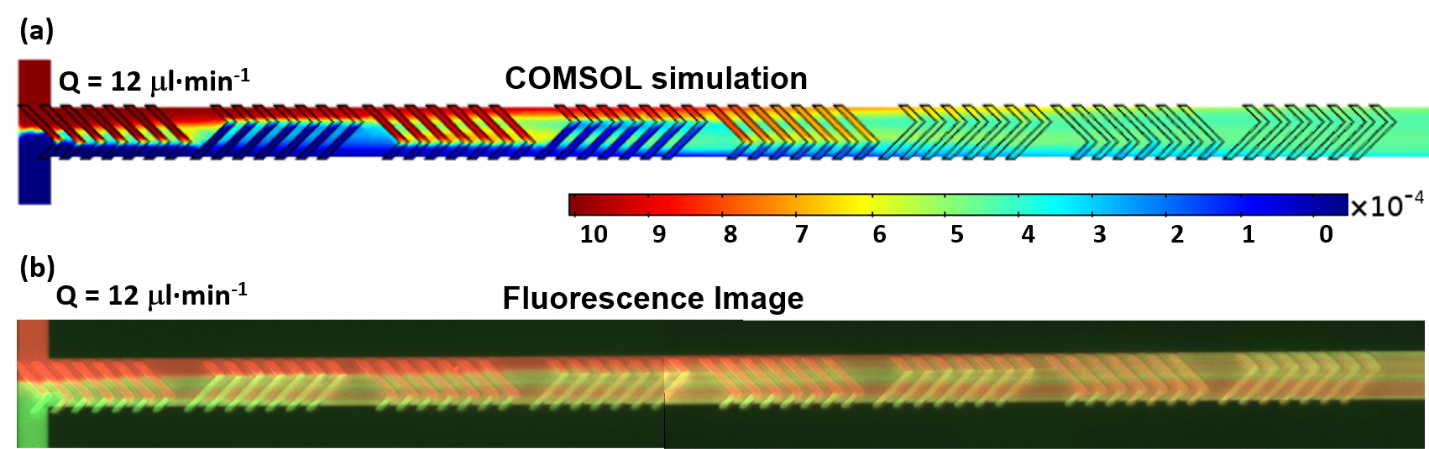


**Figure S3.** **(a)** Simulation results along the mixing channel of a classic herring-bone structure with negative grooves mixer at Q = 12µL·min^-1^ from both inlets. **(b)** Mixing of Rhodamine B conjugate 70 kDa dextran (red) and Fluorescein (green). The simulations were performed with the COMSOL Multiphyics software ([www.comsol.com](http://www.comsol.com)).

The simulation result and the experimental results of the mixing in a classic negative herring-bone channel is presented in Fig.S3. Obviously, the mixing across the channel is not simply on one direction but with a clear spiral movement. The mixing behavior had been discussed in another study[^1^](#_ENREF_1), and clearly shows that for negative SHB mixer, the homogeneous concentration distribution will only been observed after 5 or 6 circle of grooves.


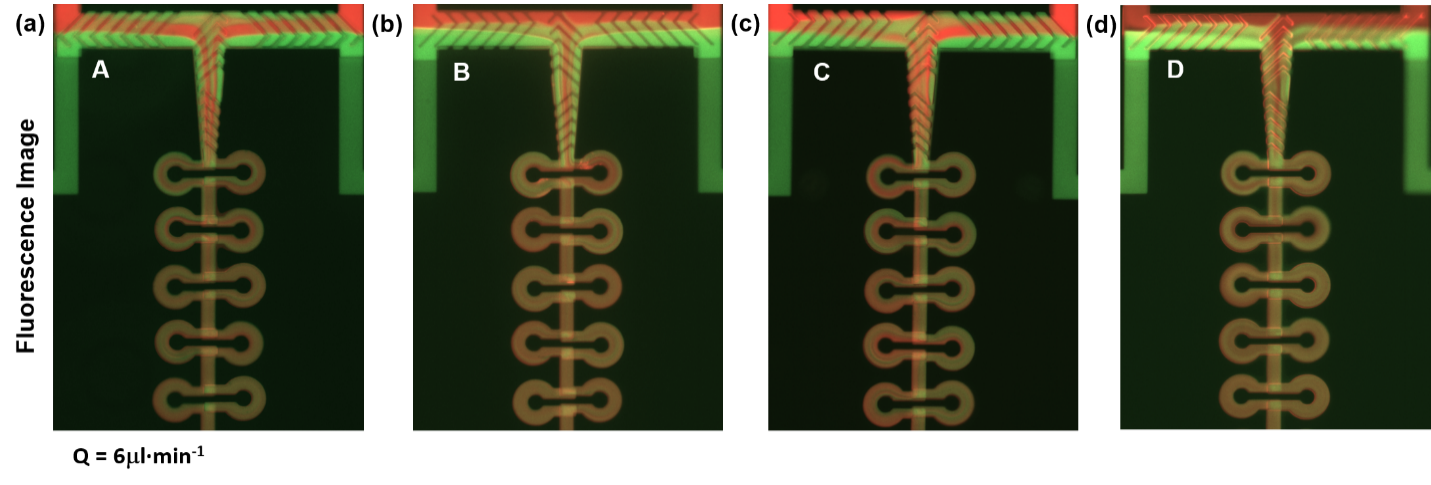


**Figure S4.** Representative fluorescence microscopy images (top: a to d) corresponding to mixing in devices with 4 positive SHB designs A, B, C, D separately. The corresponding geometry details are shown below (bottom: **a** to **d**). Flow visualization using Rhodamine B conjugate 70 kDa dextran (red) and Fluorescein (green), with the flow-rates Q for each inlet equal to 6mL·min^-1^, *Re* ~ 20 in the repeated ring-shaped units.

The fluorescence images of all four A, B, C and D positive SHB designs are shown in Fig. S4. The arrangement of the ridges can refer to the four negative SHB designs in Fig. 1. The experiment is operated under the same conditions, with the flow rates = 6 µL·min^-1^, and the concentration of the fluorescein and the Rhodamine B conjugate 70 kDa dextran are the same of the condition applied to the negative SHB designs. The geometry of the PDMS microchannel can be clearly seen and the region observed contains around 3.39 nL, which is slightly larger than the negative SHB mixer, which is 2.93 nL.

**References**

1 Kwak, T. J. *et al.* Convex Grooves in Staggered Herringbone Mixer Improve Mixing Efficiency of Laminar Flow in Microchannel. *PLOS ONE* **11**, e0166068, https://doi.org/10.1371/journal.pone.0166068 (2016).
